# Supplementary material for: Disordered RNA chaperones can enhance nucleic acid folding via local charge screening
Source: Nat Commun. 2019 Jun 5;10:2453. doi: 10.1038/s41467-019-10356-0 (PMC6549165; doi:10.1038/s41467-019-10356-0)
Supplement: Supplementary file 1 — Supplementary Information [file 41467_2019_10356_MOESM1_ESM.pdf]

**Supplementary Information for:**

# Disordered RNA chaperones can enhance nucleic acid folding via local charge screening

Erik D. Holmstrom[1,2], Zhaowei Liu[1], Daniel Nettels[1], Robert B. Best[3], and Benjamin Schuler[1,4]

[1]Department of Biochemistry, University of Zurich, Winterthurerstrasse 190, 8057 Zurich, Switzerland.
[2]Current Address: Department of Molecular Biosciences, University of Kansas, Lawrence, KS, 66045-7566, United States.
[3]Laboratory of Chemical Physics, National Institute of Diabetes and Digestive and Kidney Diseases, National Institutes of Health, Bethesda, Maryland 20892-0520, United States.
[4]Department of Physics, University of Zurich, Winterthurerstrasse 190, 8057 Zurich, Switzerland.

Correspondence and requests for materials should be addressed to E.D.H. (email: erik.d.holmstrom@ku.edu) or to R.B.B. (email: robert.best2@nih.gov) or to B.S. (email: schuler@bioc.uzh.ch)

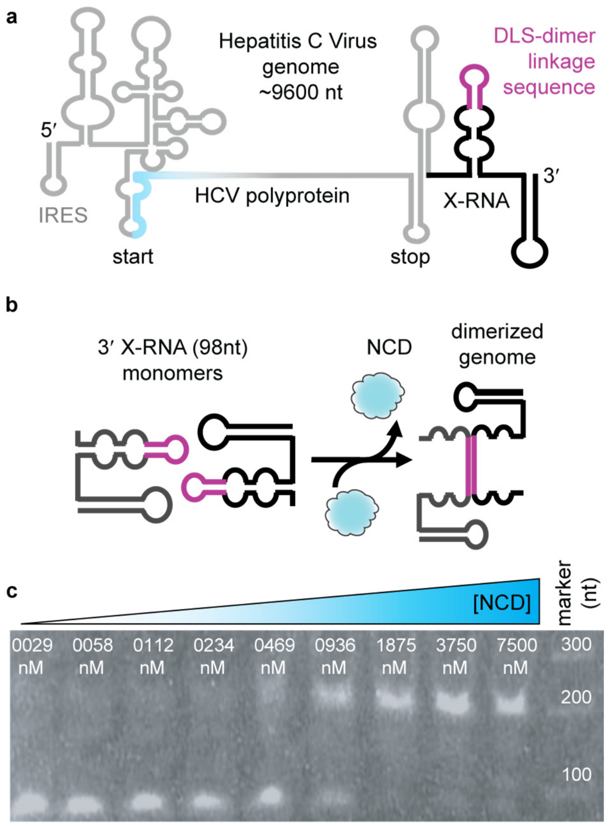


**Supplementary Figure 1. NCD dimerizes viral 3′ X-RNA**. **(a)** The hepatitis C virus genome contains a highly conserved 98-nucleotide structural element at the 3′ end called the 3′ X-RNA[1]. **(b)** This motif contains a self-complementary 16-nucleotide dimer linkage sequence (DLS). Two copies of the DLS can basepair with each other, resulting in genome dimerization. NCD acts as a nucleic acid chaperone and facilitates this process. **(c)** Electrophoretic mobility shift assay for genome dimerization. Samples containing 750 nM 3′ X-RNA were incubated for 10 minutes with NCD at 37 °C. Before the samples were loaded on the gel, NCD was digested and extracted to prevent any mobility shifts from complex formation. The original image is provided as a Source Data file.

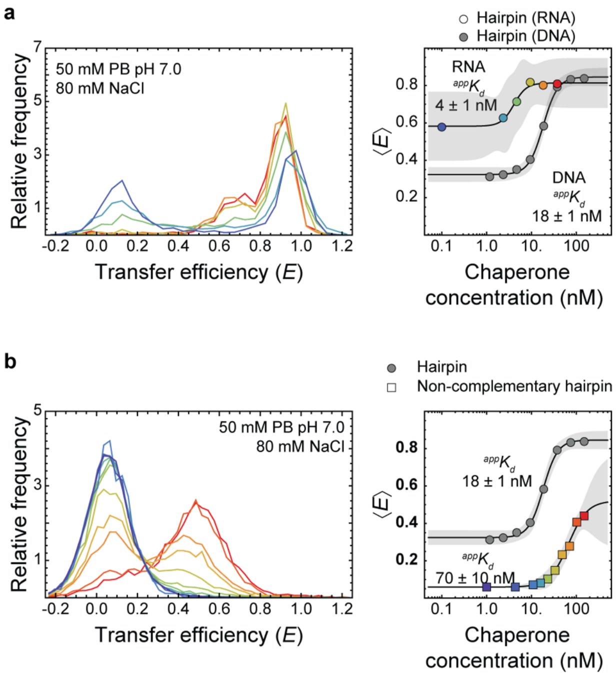


**Supplementary Figure 2. NCD binds different nucleic acids with high affinity. (a)** Free-diffusion transfer efficiency histograms (left) of the 5´-3´ FRET-labeled RNA hairpin measured at 50 mM PB pH 7.0 with 80 mM NaCl with increasing concentrations of chaperone (blue → red; see right panel for detailed color code). Mean transfer efficiencies plotted against chaperone concentration for the RNA hairpin (colored symbols) and the DNA hairpin (gray symbols) (right). **(b)** Free-diffusion transfer efficiency histograms (left) of the 5´-3´ FRET-labeled folding-incompetent (i.e., non-complementary) DNA hairpin measured at 50 mM PB pH 7.0 with 80 mM NaCl with increasing concentrations of chaperone (blue → red; see right panel for detailed color code). Mean transfer efficiencies plotted against chaperone concentration for the folding-incompetent (colored symbols) and folding competent (gray symbols) DNA hairpin (right). In both **(a and b)**, the solid lines in the mean transfer efficiency plots correspond to fits with a two-state binding isotherm, with the shaded gray regions representing 99% confidence bands. Source data for mean values are provided as a Source Data file.

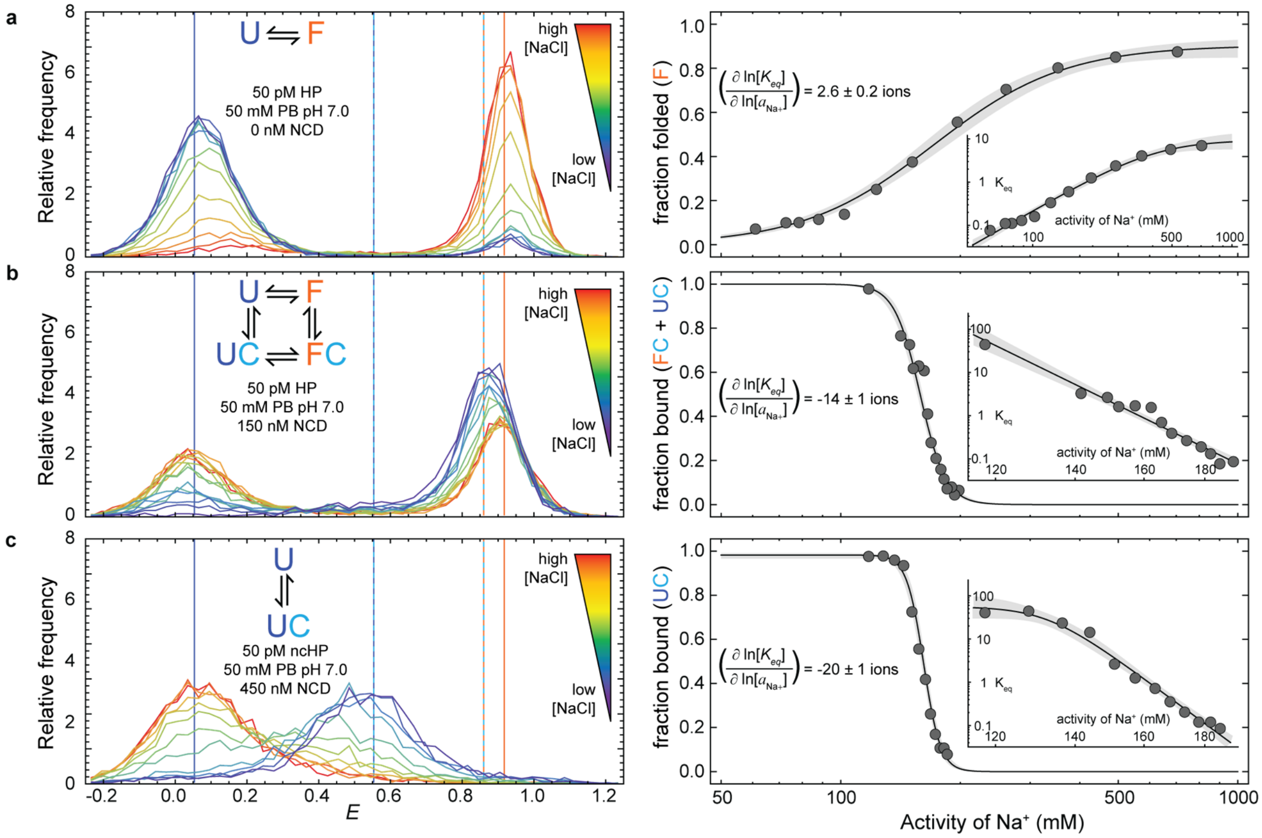


**Supplementary Figure 3. NaCl impedes chaperone-assisted folding**. Transfer efficiency histograms of freely diffusing 5´-3´ FRET-labeled hairpin at various concentrations of NaCl either **(a)** in the absence of NCD or **(b)** in the presence of 150 nM NCD. **(c)** Transfer efficiency histograms of the freely diffusing 5´-3´ FRET-labeled folding-incompetent DNA hairpin at various concentrations of NaCl in the presence of 450 nM NCD. U and F denote the unfolded and folded populations of the hairpin in the absence of chaperone, whereas UC and FC denote the unfolded and folded populations of the chaperone-bound hairpin, respectively. The right panels show how increasing the activity of $Na^+$ alters the folded **(a)** or bound **(b and c)** populations. Black lines are fits to two-state binding isotherms with 99% confidence bands shown as gray bands. Insets show the double-logarithmic dependences of the equilibrium constants on $Na^+$ activity, whose slope ($\partial \ln[K_{eq}]/\partial \ln[a_{Na+}]$) reflects the uptake or release of counter ions[2] upon folding **(a)** or binding **(b and c)**.

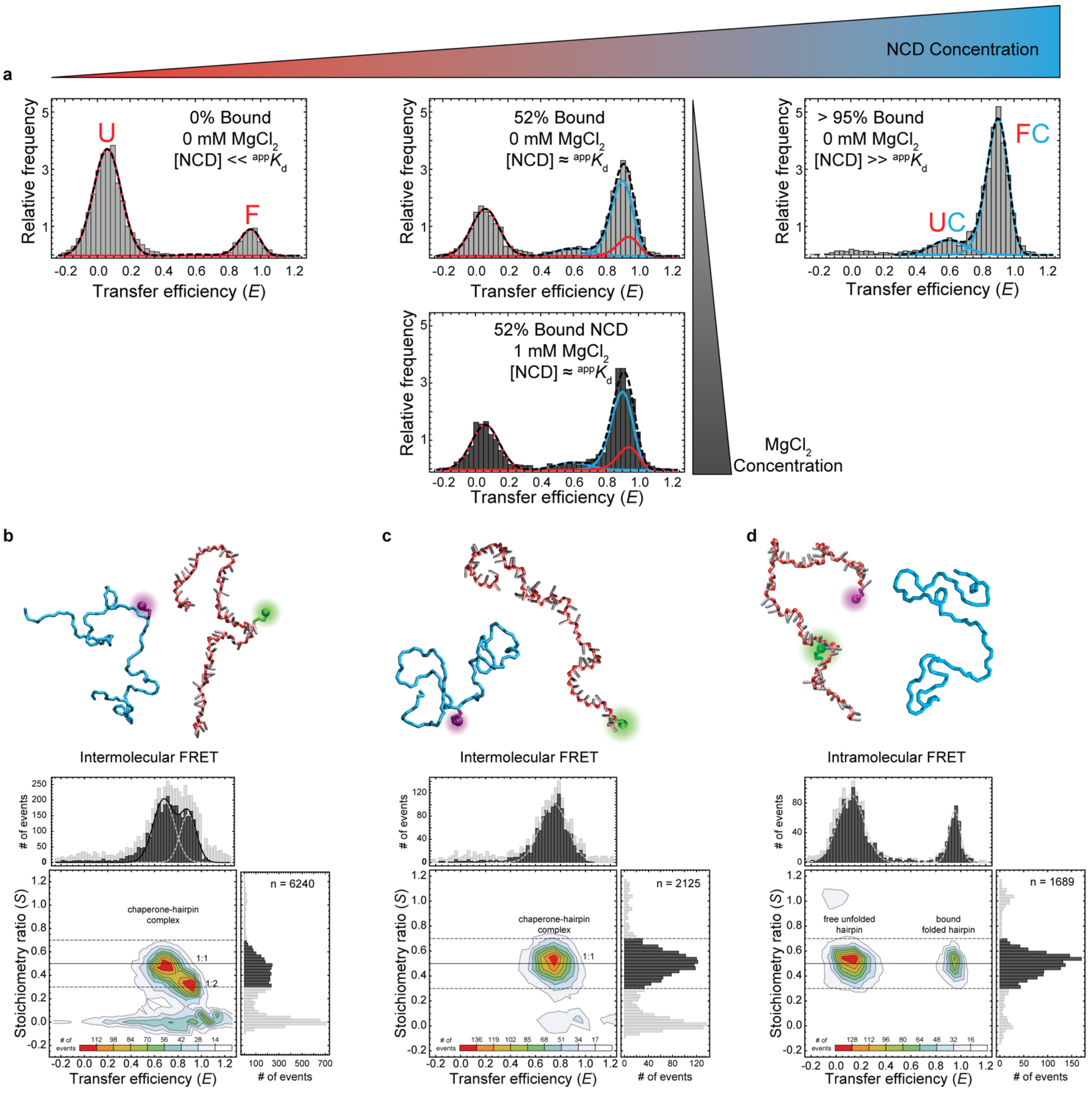


(See next page for figure caption)

**Supplementary Figure 4. Influence of divalent metal ions and example data from inter- and intramolecular FRET measurements. (a)** Addition of physiological concentrations of $MgCl_2$ does not significantly alter the fraction of bound hairpin molecules nor the transfer efficiency of the four populations (i.e., U, UC, F, and FC), suggesting that the mechanism of chaperone-assisted folding is largely unaffected by physiological concentrations of $MgCl_2$. Measurements were performed under conditions where the concentration of NCD is near the apparent dissociation constant to be maximally sensitive for changes in populations. **(b)** Pulsed interleaved excitation transfer efficiency measurements. Pulsed interleaved excitation (PIE) is used to separate FRET-labeled molecules from molecules with only a single active dye. The positions and distributions of these populations (e.g., active FRET-labeled molecules at $S \approx 1/2$, colored dark gray, as well as donor-only molecules at $S \approx 1$ and acceptor-only molecules at $S \approx 0$, colored light gray) are used to determine the correction factors (e.g., spectral cross talk, direct excitation, detection efficiency) necessary for accurate transfer efficiency measurements[3, 4, 5]. Backbone representations (top) of NCD labeled with the acceptor dye at position 65 and the donor-labeled DNA hairpin at the MID position (see Methods and Supplementary Tables 1 and 3); colors as in Figure 1a. Intermolecular transfer efficiency measurements performed in solutions containing 30 mM PB pH 7.0 with ~50 pM MID-donor-labeled DNA hairpin and ~ 250 pM T65C-acceptor-labeled NCD. The additional high-*E* population with low stoichiometry ratio (i.e., $S \approx 1/3$) indicates that these molecules have a donor-to-acceptor ratio of 1:2 and thus that two acceptor-labeled chaperones are bound to a single donor-labeled DNA molecule. However, because this work focuses on the heterodimeric nucleic acid-chaperone complex, we only consider the transfer efficiency of the 1:1 population for the structural mapping experiments (Figure 3c). **(c)** Example of intermolecular transfer efficiency measurements of the 5′donor-labeled hairpin in the presence of acceptor-labeled NCD T65C. Measurements were performed in solutions containing 30 mM PB pH 7.0 with ~ 50 pM 5′-donor-labeled DNA hairpin and ~ 125 pM T65C-acceptor-labeled NCD. Under these conditions, only the 1:1 complex is present. **(d)** Example of intramolecular transfer efficiency measurements of the 5′-3′ FRET-labeled hairpin in the presence of unlabeled NCD.

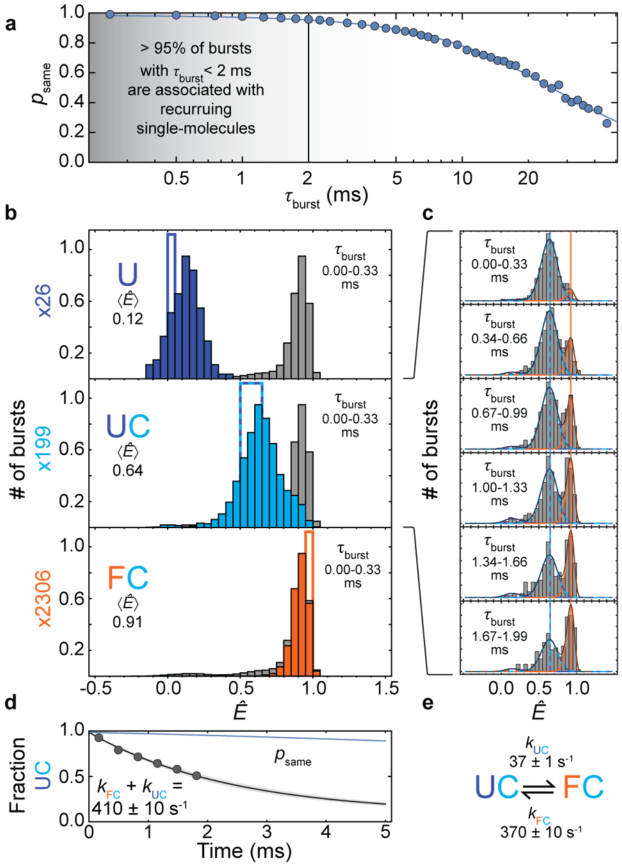


**Supplementary Figure 5. Recurrence analysis of chaperone-assisted folding. (a)** For freely diffusing single molecules, there is a finite probability, $p_{same}$, that consecutive fluorescence bursts with short inter-burst times, $\tau_{burst}$, arise from the same molecule re-entering the detection volume. This probability depends on the concentration of molecules in solution and can be determined from the auto-correlation of $\tau_{burst}$.[6] For solutions containing the 5´-3´ FRET-labeled hairpin at a concentration of ~50 pM, nearly all burst pairs separated by less than 2 ms arise from recurring molecules. The light blue line represents a fit to the data, which is used to determine $p_{same}$ as a function of $\tau_{burst}$. **(b)** Recurrence histograms of the uncorrected transfer efficiency ($\hat{E}$) are used to isolate the three most prominent populations, corresponding to the unfolded hairpin (U, blue), the chaperone-bound unfolded hairpin (UC, cyan), and the chaperone-bound folded hairpin (FC, orange), by analyzing bursts that appear less than 330 µs after the initial bursts with uncorrected transfer efficiencies between 0.0 – 0.05, 0.5 – 0.65, and 0.95 – 1.0, respectively. For comparison, the full transfer efficiency histogram, built from all photon bursts, is shown in gray. Source data for mean values are provided as a Source Data file. **(c)** Recurrence histograms with an initial $\hat{E}$ between 0.5 - 0.65 show the temporal evolution of the UC population. **(d)** As $\tau_{burst}$ increases, molecules initially in UC are more likely to recur as part of the FC population, indicating that these species interconvert on a millisecond timescale. The blue line represents $p_{same}$, and the black line is a fit to a recurrence model with 2-state interconversion; 99% confidence bands are shown in gray. **(e)** The abundance of these two populations at equilibrium is used to calculate the folding ($k_{FC}$) and unfolding ($k_{UC}$) rate constants for chaperone-assisted folding, which are consistent with the measurements of surface-immobilized molecules (Figure 2).

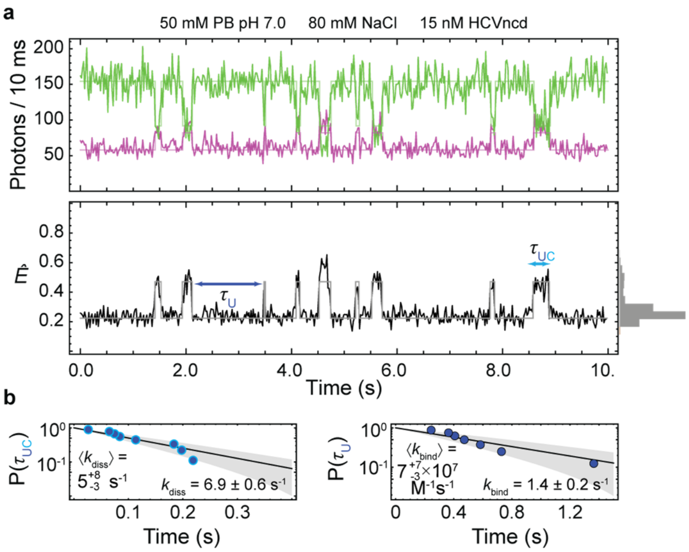


**Supplementary Figure 6. Chaperone binding and dissociation kinetics of immobilized folding-incompetent hairpin**. **(a)** Donor (light green) and acceptor (magenta) fluorescence time trace from a surface-immobilized 5´-3´ FRET-labeled folding-incompetent DNA hairpin in the presence of 15 nM NCD, with resulting uncorrected transfer efficiency (black) and state trajectory (gray) from maximum-likelihood analysis. **(b)** The distribution of dwell times in the bound ($\tau_{UC}$) and free states ($\tau_U$) from the state trajectory are fit with single-exponential decays to determine the apparent binding and dissociation rate constants, ${}^{app}k_{bind}$ and ${}^{app}k_{diss}$, respectively. $\langle {}^{app}k_{bind} \rangle$ and $\langle {}^{app}k_{diss} \rangle$ represent the mean values obtained from 27 single-molecule time traces. Source data for mean values are provided as a Source Data file. The black lines in **(b)** are single-exponential fits with 99% confidence bands shown in gray.

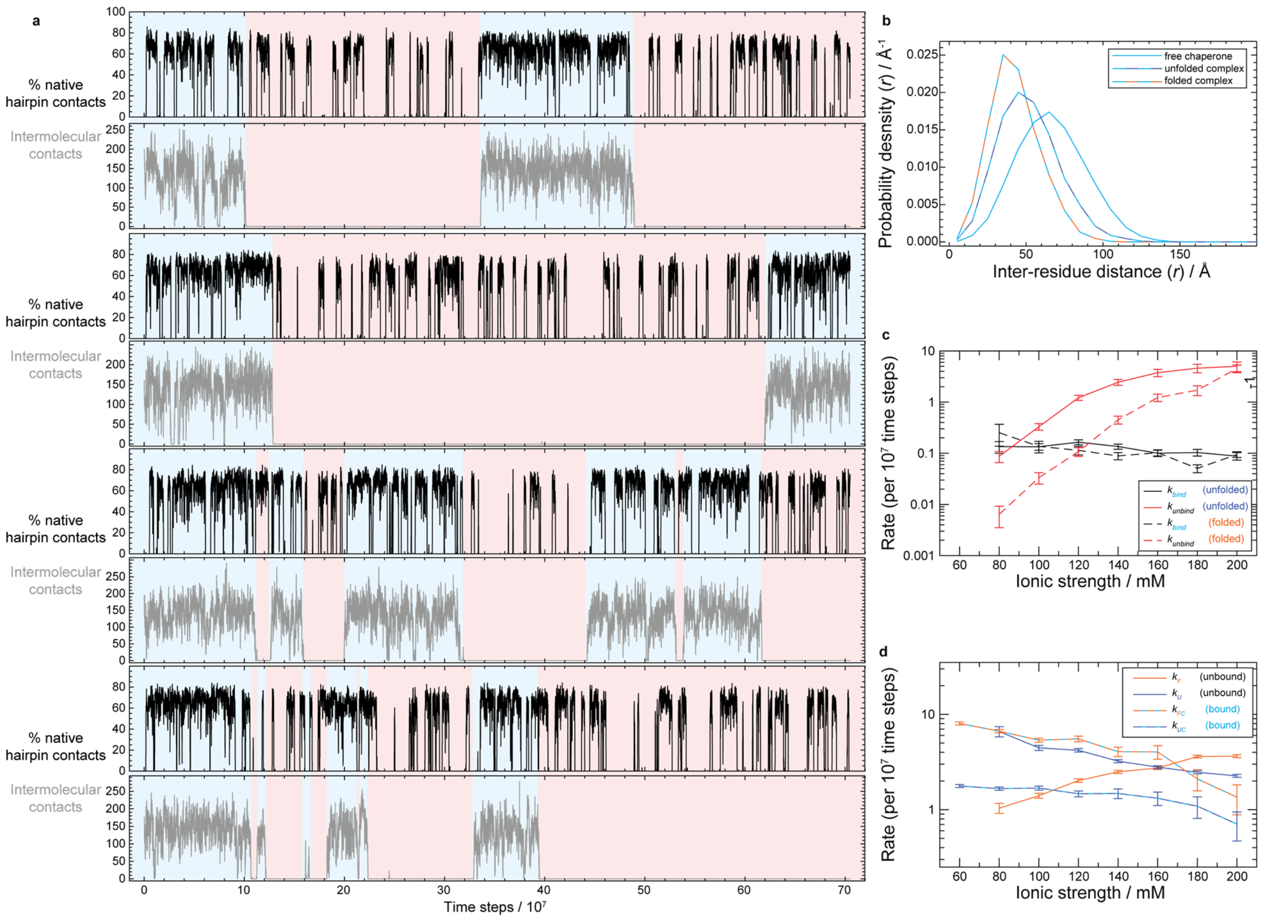

**Supplementary Figure 7. Coarse-grained molecular dynamics simulations. (a)** Sample trajectories from coarse-grained molecular dynamics simulations of the nucleic acid and chaperone with a Debye screening term that corresponds to an ionic strength of 120 mM (see Supplementary Movie 1 and 2). The gray trajectories show the total number of intermolecular contacts between the nucleic acid hairpin and the chaperone. The black trajectories display the percentage of native contacts (Q) within the nucleic acid hairpin, which are more likely to be formed when bound to the chaperone. Time trajectories display regular transitions between chaperone-free (red shading) and chaperone-bound kinetic regimes (cyan shading). **(b)** Distribution of intramolecular distances from the coarse-grained molecular dynamics simulations measured between position 2 and 65 of the chaperone, corresponding to the FRET-labeled NCD S2C-T65C. The distance distributions are broad owing to the lack of structure in the chaperone, and binding to either the folded or unfolded hairpin gives rise to compaction. **(c)** and **(d)** Kinetic parameters from simulations (see Methods) where the Debye length is adjusted to approximate the effects of charge screening at various ionic strengths. Note that the reported values of ionic strength cannot be quantitatively compared to the experimental ionic strengths, because the coarse-grained model does not consider ions explicitly.

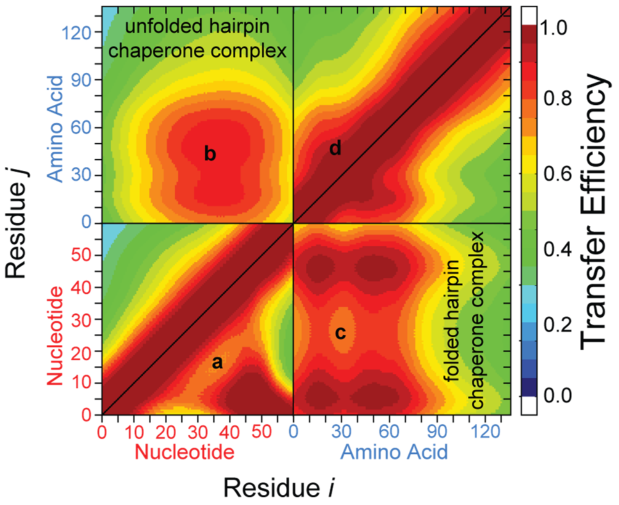


**Supplementary Figure 8. Transfer efficiency map from coarse-grained molecular dynamics simulations**. Regions from simulation trajectories corresponding to the unfolded hairpin-chaperone complex (UC; above the diagonal) and the folded hairpin-chaperone complex (FC; below the diagonal) were analyzed to determine the mean transfer efficiencies associated with the distance distributions of all possible pairs of residues. Intermolecular transfer efficiencies of UC and FC are shown in the upper left and bottom right, respectively, Intramolecular transfer efficiencies of UC are shown above the diagonal in the top right (chaperone) and bottom left (hairpin) quadrants. Intramolecular transfer efficiencies of FC are shown below the diagonal in the top right (chaperone) and bottom left (hairpin) **(a)** Nucleotides 1-7 are able to form a hairpin with nucleotides 48-54 when bound to the chaperone. **(b)** The N-terminal half of NCD, containing two patches of positively charged amino acids (residues 6-23 and 39-62), is on average closest to the center of the unfolded hairpin. **(c)** The same positively charged segment of NCD interacts most strongly with the stem-loop junction (nucleotides 7-8 and 47-48) in the folded hairpin. **(d)** The intramolecular dimensions of the chaperone are largely unaffected by the folding of the hairpin.

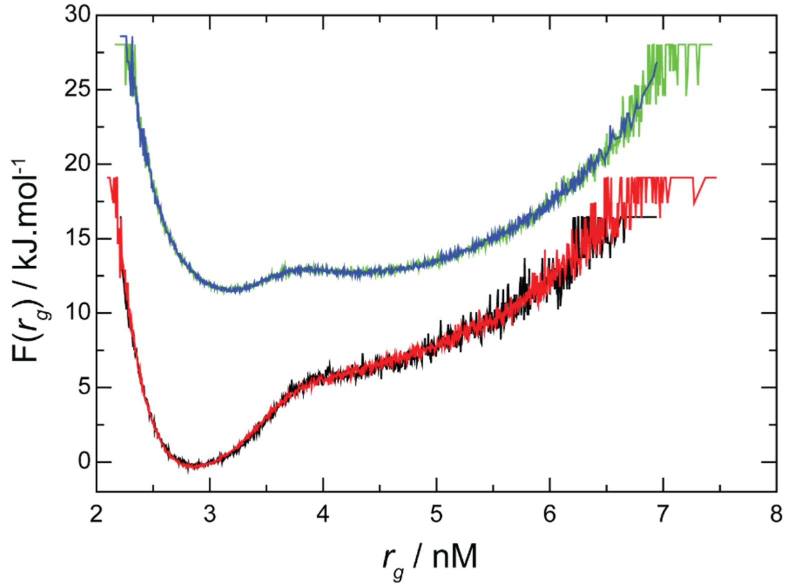


| | (per $10^7$ time steps) $k_{fold}$ | $k_{unfold}$ |
|---|---|---|
| DNA/protein unbound | 2.0 ± 0.1 | 4.2 ± 0.2 |
| DNA/protein bound | 5.5 ± 0.4 | 1.5 ± 0.1 |
| DNA only (no bias) | 1.9 ± 0.1 | 3.9 ± 0.2 |
| DNA only ($r_g$ bias) | 8.7 ± 0.3 | 3.1 ± 0.1 |
| DNA only ($r_g$ bias) scaled | 5.2 ± 0.2 | 1.8 ± 0.1 |

**Supplementary Figure 9. Nucleic acid chain compaction increases hairpin folding rate constant in simulations**. Placing the DNA in an external potential chosen to bias the radius of gyration ($r_g$) to match that of the chaperone-bound DNA shifts the equilibrium to favor hairpin formation. The change in equilibrium constant is predominantly caused by an increase in the folding rate. Experimentally, chaperone binding also gives rise to a shift in the equilibrium constant that favors folding, resulting primarily from an increase in the folding rate constant (Figure 2). Comparisons among these different simulations reveal that the hairpin folding and unfolding rates with the $r_g$ bias (red) are the same as those when NCD is bound (black), except for a scaling factor of ~ 0.592, which is presumably due to the additional internal friction in the nucleoprotein complex caused by the presence of NCD.

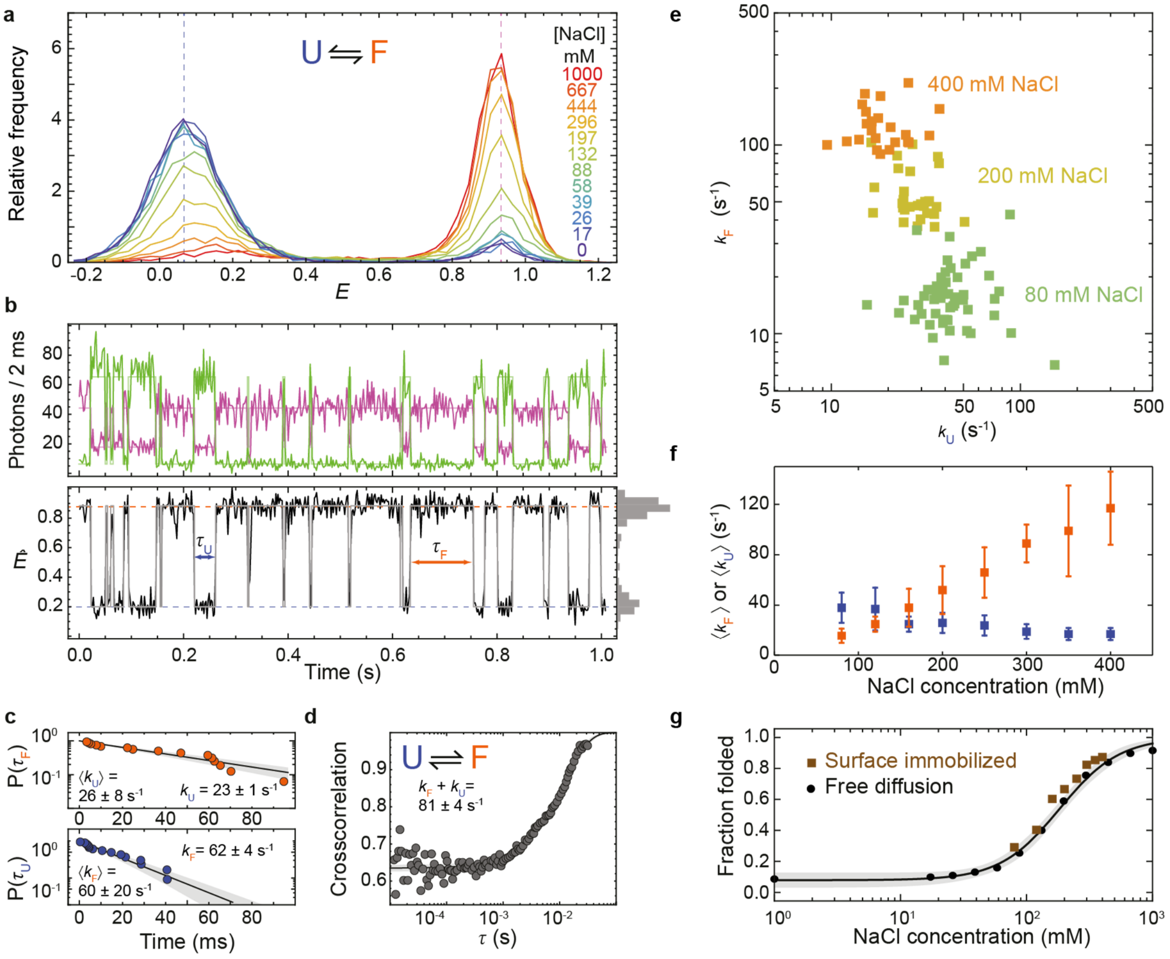


(See next page for figure caption)

**Supplementary Figure 10. Equilibrium and kinetic analysis of NaCl-dependent hairpin folding via FRET**. **(a)** Transfer efficiency histograms of freely diffusing 5′-3′ FRET-labeled hairpin at various concentrations of NaCl. The peak at low $E$ corresponds to the unfolded population of the hairpin (U, blue), whereas the peak at high $E$ results from folded hairpin molecules (F, orange). **(b)** Donor (green) and acceptor (magenta) fluorescence time trace from a surface-immobilized 5′-3′ FRET-labeled hairpin at 200 mM NaCl in 50 mM sodium phosphate buffer pH 7.0. This information is used to generate both the uncorrected transfer efficiency ($\widehat{E}$, black) and the Viterbi-based state trajectory (gray) for a two-state Markov model. **(c)** The survival probability distribution based on dwell times in the folded (top) and unfolded (bottom) segments of the state trajectory are fit to single-exponential decays to determine the unfolding and folding rate constants, $k_U$ and $k_F$, respectively; mean values, $\langle k_U \rangle$ and $\langle k_F \rangle$, were obtained from 30 single-molecule measurements, with an uncertainty corresponding to their standard deviation. Source data for mean values are provided as a Source Data file. **(d)** Donor-acceptor cross-correlation for interphoton times, $\tau$, from 0.01 ms to 100 ms; decorrelation of the anti-correlated signal occurs with a rate constant that is consistent with the dwell-time analysis. **(e)** Scatter plot showing the distribution of $k_F$ and $k_U$ from individual single-molecule measurements at different NaCl concentrations. **(f)** Plot of $\langle k_F \rangle$ and $\langle k_U \rangle$ as a function of NaCl concentration; error bars reflect the standard deviations of the measurements. Source data for mean values are provided as a Source Data file. **(g)** Comparison of the fractional abundance of the folded population for experiments with surface-immobilized molecules and in free diffusion as a function of NaCl concentration. The agreement between the two data sets suggests that surface immobilization does not alter the equilibrium behavior of the hairpin substantially. Solid black lines represent fits with either a single-exponential decay **(c and d)** or a two-state binding isotherm **(g)**; in all cases, 99% confidence bands are shown in gray.

## Supplementary Table 1

| | |
|---|---|
| 5′-3′ | 5′/5Thio/CTCTTCAAAAAAAAAAAAAAAAAAAACCAAAAAAAAAAAAAAAAAAA/*t*/GAAGAGAAAAAA/3Bio/3′ |
| 5′-MID | 5′/5Thio/CTCTTCAAAAAAAAAAAAAAAAAAAAC/*t*/AAAAAAAAAAAAAAAAAAATGAAGAGAAAAAA/3Bio/3′ |
| 3′-MID | 5′/CTCTTCAAAAAAAAAAAAAAAAAAAAC/*t*/AAAAAAAAAAAAAAAAAAA/*t*/GAAGAGAAAAAA/3Bio/3′ |
| MID | 5′/CTCTTCAAAAAAAAAAAAAAAAAAAAC/*t*/AAAAAAAAAAAAAAAAAAATGAAGAGAAAAAA/3Bio/3′ |
| nc-5′-3′ | 5′/5Thio/GAGAAGTAAAAAAAAAAAAAAAAAAACCAAAAAAAAAAAAAAAAAAA/*t*/GAAGAGAAAAAA/3Bio/3′ |
| nc-5′-MID | 5′/5Thio/GAGAAGTAAAAAAAAAAAAAAAAAAAC/*t*/AAAAAAAAAAAAAAAAAAATGAAGAGAAAAAA/3Bio/3′ |
| nc-3′-MID | 5′/GAGAAGTAAAAAAAAAAAAAAAAAAAC/*t*/AAAAAAAAAAAAAAAAAAA/*t*/GAAGAGAAAAAA/3Bio/3′ |
| nc-MID | 5′/GAGAAGTAAAAAAAAAAAAAAAAAAAC/*t*/AAAAAAAAAAAAAAAAAAATGAAGAGAAAAAA/3Bio/3′ |

Table of oligonucleotide sequences for the DNA hairpin and the non-complementary DNA hairpin. Underlined nucleotides represent nucleotides located in the stem of the hairpin.

/5Thio/ – 5′ dithiol at position for maleimide labeling
/*t*/ – internal amino-modified C6 dT for succinimidyl ester labeling
/3Bio/ – 3′ biotin with a 6-carbon spacer for surface immobilization

**Supplementary Table 2**

| | |
|---|---|
| 5′-3′ (RNA) | 5′/5Thio/G<u>UCUUCA</u>AAAAAAAAAAAAAAAAAAACCAAAAAAAAAAAAAAAAAAA/<u>*t*</u>/<u>GAAGA</u>GAAAAAA/3Bio/3′ |

Table of the oligonucleotide sequence of the RNA hairpin. Underlined nucleotides represent nucleotides located in the stem of the hairpin.

/<u>5Thio</u>/ – 5′ dithiol at position for maleimide labeling
/*t*/ – internal amino-modified dT for succinimidyl ester labeling
/3Bio/ – 3′ biotin with a 6 carbon atom spacer for surface immobilization

**Supplementary Table 3**

| | |
|---|---|
| CAE46584.1 | 000 MSTNPKPQRK 010 TKRNTNRRPQ 020 DVKFPGGGQI 030 VGGVYLLPRR 040 GPRLGVRATR<br>050 KTSERSQPRG 060 RRQPTPRHVG 070 PRAGPGLSPG 080 TLGPSMAMRA 090 AVGRDGSCLP<br>100 VALGLAGAPQ 110 TPGVGRAIWV 120 RSSIPLRAAS 130 PTSW |
| S2C | PGP 000 MCTNPKPQRK 010 TKRNTNRRPQ 020 DVKFPGGGQI 030 VGGVYLLPRR 040 GPRLGVRATR<br>050 KTSERSQPRG 060 RRQPTPRHVG 070 PRAGPGLSPG 080 TLGPSMAMRA 090 AVGRDGSMLP<br>100 VALGLAGAPQ 110 TPGVGRAIWV 120 RSSIPLRAAS 130 PTS |
| T65C | PGP 000 MSTNPKPQRK 010 TKRNTNRRPQ 020 DVKFPGGGQI 030 VGGVYLLPRR 040 GPRLGVRATR<br>050 KTSERSQPRG 060 RRQPCPRHVG 070 PRAGPGLSPG 080 TLGPSMAMRA 090 AVGRDGSMLP<br>100 VALGLAGAPQ 110 TPGVGRAIWV 120 RSSIPLRAAS 130 PTS |
| S130C | PGP 000 MSTNPKPQRK 010 TKRNTNRRPQ 020 DVKFPGGGQI 030 VGGVYLLPRR 040 GPRLGVRATR<br>050 KTSERSQPRG 060 RRQPTPRHVG 070 PRAGPGLSPG 080 TLGPSMAMRA 090 AVGRDGSMLP<br>100 VALGLAGAPQ 110 TPGVGRAIWV 120 RSSIPLRAAC 130 PTS |
| S2C-S130C | PGP 000 MCTNPKPQRK 010 TKRNTNRRPQ 020 DVKFPGGGQI 030 VGGVYLLPRR 040 GPRLGVRATR<br>050 KTSERSQPRG 060 RRQPTPRHVG 070 PRAGPGLSPG 080 TLGPSMAMRA 090 AVGRDGSMLP<br>100 VALGLAGAPQ 110 TPGVGRAIWV 120 RSSIPLRAAC 130 PTS |
| S2C-T65C | PGP 000 MCTNPKPQRK 010 TKRNTNRRPQ 020 DVKFPGGGQI 030 VGGVYLLPRR 040 GPRLGVRATR<br>050 KTSERSQPRG 060 RRQPCPRHVG 070 PRAGPGLSPG 080 TLGPSMAMRA 090 AVGRDGSMLP<br>100 VALGLAGAPQ 110 TPGVGRAIWV 120 RSSIPLRAAS 130 PTS |
| T65C-S130C | PGP 000 MSTNPKPQRK 010 TKRNTNRRPQ 020 DVKFPGGGQI 030 VGGVYLLPRR 040 GPRLGVRATR<br>050 KTSERSQPRG 060 RRQPCPRHVG 070 PRAGPGLSPG 080 TLGPSMAMRA 090 AVGRDGSMLP<br>100 VALGLAGAPQ 110 TPGVGRAIWV 120 RSSIPLRAAC 130 PTS |

Table of amino acid sequences of all NCD variants used in this study.

**Supplementary Table 4**

| **Bonds** | **$r_0$ (nm)** | **$k_b$ (kJ mol$^{-1}$ nm$^{-2}$)** |
|---|---|---|
| $C_i - C_{i+1}$ | 0.381 | 300,000 |
| $B_i - R_i$ | 0.586 | 300,000 |
| $P_i - R_i$ | 0.390 | 300,000 |
| $P_i - R_{i+1}$ | 0.390 | 300,000 |
| **Angles** | **$\theta_0$ (degrees)** | **$k_\theta$ (kJ mol$^{-1}$ rad$^{-2}$)** |
| $C_i - C_{i+1} - C_{i+2}$ | 130.0 | 600.0 |
| $B_i - R_i - P_i$ | 100.8 | 1000.0 |
| $P_i - R_{i+1} - B_{i+1}$ | 91.1 | 1000.0 |
| $R_i - P_i - R_{i+1}$ | 95.2 | 1000.0 |

Table of ideal bond lengths and angles, and corresponding force constants for the model. All ideal bond lengths and angles were taken from canonical structures. No bonded parameters were optimized.

**Supplementary Table 5**

| **Pair type** | $\sigma$ **(nm)** | $\varepsilon$ **(kJ mol$^{-1}$)** |
|---|---|---|
| *Protein-Protein* | | |
| $C_i - C_j$ | 0.600 | 0.377 |
| *DNA-DNA* | | |
| $B_i - B_{i+1}$ (i.e. stacking) | 0.360 | 8.786 |
| $B_i - B_j$ | 0.600 | 0.100 |
| $P_i - P_j$ | 0.600 | 0.100 |
| $R_i - R_j$ | 0.600 | 0.100 |
| $B_i - R_j$ | 0.600 | 0.100 |
| $B_i - P_j$ | 0.600 | 0.100 |
| $R_i - P_j$ | 0.600 | 0.100 |
| *Protein-DNA* | | |
| $B_i - C_j$ | 0.600 | 0.151 |
| $P_i - C_j$ | 0.600 | 0.151 |
| $R_i - C_j$ | 0.600 | 0.151 |

Table of non-bonded parameters. Colored parameters were optimized to match FRET data; parameters with the same color were linked in the parameter optimization.

**Supplementary Table 6**

| **Pair type** | $\sigma$ **(nm)** | $\varepsilon$ **(kJ.mol$^{-1}$)** |
|---|---|---|
| $B_i - B_j$ | 0.359 | 3.279* |
| $B_i - B_{j-1}$ | 0.424 | 3.279* |
| $B_i - R_{j-1}$ | 0.808 | 3.279* |
| $B_i - B_{j-2}$ | 0.677 | 3.279* |
| $B_i - R_{j-2}$ | 0.817 | 3.279* |
| $B_i - B_{j+1}$ | 0.536 | 3.279* |
| $B_i - B_{j+2}$ | 0.804 | 3.279* |

Table of native contacts. Native contacts occur when residues *i* and *j* are base-paired (colored parameters were linked in parameter optimization). Note that, by symmetry, analogous native contacts are implicitly defined with *j* and *i* permuted. The native contact distances are based on idealized B-form Watson-Crick DNA.

*The contact energies listed are those used for simulating equilibrium folding dynamics of the DNA; for simulating the folded state, a value of 5.0 kJ mol$^{-1}$ was used to keep the DNA folded.

## Supplementary Table 7

| | |
|---|---|
| HCVcpv2/TGC->TCC#1 | GGGACCCGGGATGTCCACCAACCCGAAACCGCAGCGTAAG |
| HCVcpv2/TGC->TCC#2 | GTTTCGGGTTGGTGGACATCCCGGGTCCCTGAAAGAGGAC |
| HCVcpv2/TGT->CAT#1 | GCGTGGCCGTCGTCAGCCGCATCCGCGTCATGTTGGC |
| HCVcpv2/TGT->CAT#2 | CACGCGGGCCAACATGACGCGGATGCGGCTGACGACG |
| C2S-forward-v2 | GGGATGTCCACCAACCCGAAACCGCAGC |
| C2S-reverse-v2 | GTTGGTGGACATCCCGGGTCCCTGAAAGAG |
| C65H-forward-v2 | CAGCCGCATCCGCGTCATGTTGGCCCG |
| C65H-reverse-v2 | CGCGGATGCGGCTGACGACGGCCAC |
| C65T-forward | CAGCCGACTCCGCGTCATGTTGGCCCG |
| C65T-reverse | CGCGGAGTCGGCTGACGACGGCCAC |
| S130C-forward | GCTGCGTGTCCGACTTCTTAATTAACCTAGGCTGC |
| S130C-reverse | GTCGGACACGCAGCACGCAGCGGGATAG |

Complete list of all oligonucleotide primers used in this study

**Supplementary References**


1. Ivanyi-Nagy R*, et al.* Analysis of hepatitis C virus RNA dimerization and core-RNA interactions. *Nucleic Acids Research* **34**, 2618-2633 (2006).

2. Record MT, Jr., Anderson CF, Lohman TM. Thermodynamic analysis of ion effects on the binding and conformational equilibria of proteins and nucleic acids: the roles of ion association or release, screening, and ion effects on water activity. *Quarterly Reviews of Biophysics* **11**, 103-178 (1978).

3. Lee NK*, et al.* Accurate FRET measurements within single diffusing biomolecules using alternating-laser excitation. *Biophysical Journal* **88**, 2939-2953 (2005).

4. Kudryavtsev V, Sikor M, Kalinin S, Mokranjac D, Seidel CA, Lamb DC. Combining MFD and PIE for accurate single-pair Forster resonance energy transfer measurements. *ChemPhysChem* **13**, 1060-1078 (2012).

5. Holmstrom ED, Holla A, Zheng W, Nettels D, Best RB, Schuler B. Accurate Transfer Efficiencies, Distance Distributions, and Ensembles of Unfolded and Intrinsically Disordered Proteins From Single-Molecule FRET. *Methods in enzymology* **611**, 287-325 (2018).

6. Hoffmann A*, et al.* Quantifying heterogeneity and conformational dynamics from single molecule FRET of diffusing molecules: recurrence analysis of single particles (RASP). *Physical Chemistry Chemical Physics* **13**, 1857-1871 (2011).
