# Supplementary material for: Disordered RNA chaperones can enhance nucleic acid folding via local charge screening
Source: Nat Commun. 2019 Jun 5;10:2453. doi: 10.1038/s41467-019-10356-0 (PMC6549165; doi:10.1038/s41467-019-10356-0)
Supplement: Supplementary file 6 — Reporting Summary [file 41467_2019_10356_MOESM6_ESM.pdf]

nature research

Corresponding author(s): Erik D. Holmstrom
Benjamin Schuler
Robert Best

Last updated by author(s): Mar 22, 2019

Reporting Summary

Nature Research wishes to improve the reproducibility of the work that we publish. This form provides structure for consistency and transparency in reporting. For further information on Nature Research policies, see Authors & Referees and the Editorial Policy Checklist.

Statistics

For all statistical analyses, confirm that the following items are present in the figure legend, table legend, main text, or Methods section.

n/a	Confirmed	
☐	☒	The exact sample size (*n*) for each experimental group/condition, given as a discrete number and unit of measurement
☐	☒	A statement on whether measurements were taken from distinct samples or whether the same sample was measured repeatedly
☒	☐	The statistical test(s) used AND whether they are one- or two-sided<br>*Only common tests should be described solely by name; describe more complex techniques in the Methods section.*
☒	☐	A description of all covariates tested
☒	☐	A description of any assumptions or corrections, such as tests of normality and adjustment for multiple comparisons
☐	☒	A full description of the statistical parameters including central tendency (e.g. means) or other basic estimates (e.g. regression coefficient) AND variation (e.g. standard deviation) or associated estimates of uncertainty (e.g. confidence intervals)
☒	☐	For null hypothesis testing, the test statistic (e.g. *F*, *t*, *r*) with confidence intervals, effect sizes, degrees of freedom and *P* value noted<br>*Give P values as exact values whenever suitable.*
☒	☐	For Bayesian analysis, information on the choice of priors and Markov chain Monte Carlo settings
☒	☐	For hierarchical and complex designs, identification of the appropriate level for tests and full reporting of outcomes
☒	☐	Estimates of effect sizes (e.g. Cohen's *d*, Pearson's *r*), indicating how they were calculated

Our web collection on statistics for biologists contains articles on many of the points above.

Software and code

Policy information about availability of computer code

Data collection	All data collection requiring software was collected using either PicoQuant SymphoTime 64; Simulations were run with standard Gromacs, versions 5.1.4, available from www.gromacs.org. For the biased simulations, gromacs was patched with plumed 2.4.3, available from www.plumed.org
Data analysis	A custom module for Mathematica 11 (Wolfram Research) used for the analysis of single-molecule fluorescence data is also available upon request.

For manuscripts utilizing custom algorithms or software that are central to the research but not yet described in published literature, software must be made available to editors/reviewers. We strongly encourage code deposition in a community repository (e.g. GitHub). See the Nature Research guidelines for submitting code & software for further information.

Data

Policy information about availability of data

All manuscripts must include a data availability statement. This statement should provide the following information, where applicable:

- Accession codes, unique identifiers, or web links for publicly available datasets
- A list of figures that have associated raw data
- A description of any restrictions on data availability

Data supporting the findings of this manuscript are available from the corresponding authors upon reasonable request. The source data underlying mean values and gel images in Figure panels 1c, 2c, 2d, 3c, 4c, 4d, S1c, S2a, S2b, S5b, S6b, S10c, and S10f are provided as a Source Data file.

Field-specific reporting

Please select the one below that is the best fit for your research. If you are not sure, read the appropriate sections before making your selection.

☒ Life sciences ☐ Behavioural & social sciences ☐ Ecological, evolutionary & environmental sciences

For a reference copy of the document with all sections, see nature.com/documents/nr-reporting-summary-flat.pdf

Life sciences study design

All studies must disclose on these points even when the disclosure is negative.

Sample size	The sample size for all free diffusion measurements was always > 1000 bursts. The sample size for all surface immobilized measurements was always > 20 individual molecules. The length of the simulations was at least 500 times the typical distance correlation time within folded or unfolded states, corresponding to ~500 independent samples within each state. Folding and unfolding rates were computed from a minimum (although usually many more) of 10 events each.
Data exclusions	Freely diffusing burst arising from molecules with only a single fluorophore and with < 50 photons were excluded from analysis. Surface immobilized time traces were inspected to ensure that no substantial brightness variations were occurring (e.g., drift of the molecule's position, long-lived dark states, or background fluctuations). Single-step photobleaching indicated that only one molecule was present in the confocal volume. All time traces with < 10 conformational transitions were excluded from analysis. For the simulations, the first half of each trajectory was discarded as equilibration.
Replication	The overall reproducibility of the experiments was confirmed by making multiple measurements of newly prepared samples under slightly different experimental conditions to identify how the experimental observable varied with the experimental variable.
Randomization	No randomization was used.
Blinding	Experiments were not blinded.

Reporting for specific materials, systems and methods

We require information from authors about some types of materials, experimental systems and methods used in many studies. Here, indicate whether each material, system or method listed is relevant to your study. If you are not sure if a list item applies to your research, read the appropriate section before selecting a response.

Materials & experimental systems

n/a	Involved in the study	
☒	☐	Antibodies
☒	☐	Eukaryotic cell lines
☒	☐	Palaeontology
☒	☐	Animals and other organisms
☒	☐	Human research participants
☒	☐	Clinical data

Methods

n/a	Involved in the study	
☒	☐	ChIP-seq
☒	☐	Flow cytometry
☒	☐	MRI-based neuroimaging
