# Supplementary material for: Disordered RNA chaperones can enhance nucleic acid folding via local charge screening
Source: Nat Commun. 2019 Jun 5;10:2453. doi: 10.1038/s41467-019-10356-0 (PMC6549165; doi:10.1038/s41467-019-10356-0)
Supplement: Supplementary file 7 — Description of Additional Supplementary Files [file 41467_2019_10356_MOESM7_ESM.docx]

**Title: Supplementary Movie 1. Simulation of folding and binding of DNA with NCD.**
**Description:** The movie shows a short fragment of a simulation in which multiple folding and unfolding events are observed. The DNA backbone (phosphate, ribose) is colored red with the bases in white, except for residues forming base pairs in the native duplex, which are represented in orange. NCD is shown in blue. The two graphs below show the evolution of the fraction of native contacts, Q, in red, and the number of intermolecular contacts in blue as the simulation progresses. Note the increased probability of hairpin formation (high Q values) when NCD is bound (large number of contacts). For ease of visualization, the trajectory has been aligned by least squares to the native duplex, using the orange residues. The trajectory has also been smoothed to reduce jitter by averaging the coordinates of adjacent frames.

**Title: Supplementary Movie 2. Zoomed-in trajectory showing a single folding event of DNA while bound to NCD at high time resolution.**
**Description:** Representation of both molecules is the same as in Movie S1, and fraction of native contacts, Q, for the DNA is shown below in red. In this case, the trajectory was not aligned to a reference structure, and no coordinate smoothing was performed.

**Title: Supplementary Software
Description:** Example input files for running the coarse-grained model in Gromacs (www.gromacs.org) are provided in a compressed archive (containing initial coordinates, topology files, run input and auxiliary files, and a summary README file) as a Supplementary file (Simulation_Models.tgz).
